# The Natural History of Cancer

**Published:** 1905-06

**Authors:** 


### THE NATURAL HISTORY OF CANCER.

THE origin of cancer, after years of careful and exhaustive research and orientalism, still remains one of the mysteries of medicine. Theories of its genesis are brought forward from time to time, attract some attention and vanish. There has been but a trifling gain in our knowledge of the etiology of this ever broadening road to immortality in the memory of man.

W. A. Freund quoted in the *Journal of the American Medical Association* for April 8, regards senilism in the widest sense of the term as the prime etiological factor in cancer. The senile changes may be premature or timely, and they may be localized, affecting skin, glands and mucosa, or they may be generalized ; occur spontaneously and normally in these parts of the body, or abnormally. An indolent degenerative process, with mild and insignificant symptoms may suddenly take on an acutely malignant phase. Promising field of research lies in the study of the parts most frequently affected by cancer before the growth appears, and also in the conduct of cells of various adjacent tissues separated from their environment. Wentscher has conducted research on the persistent vitality of cells of the rete malpighii in detached scraps of skin.

Freund believes depressing influences have an effect upon determining the transition of a "borderland" growth into a malignant one. As to curability, Freund thinks that permanent cures follow early destruction of primary carcinomata, especially those occurring about the face and tumors developing from warts and moles. Metastatsic growths are more amenable to treatment than the original tumors. Spare, elderly patients have better prospects than younger and stouter ones, especially pregnant women. Spontaneous retrogression of metastases may follow removal of the primary growth. Freund emphasizes the importance of hygiene, of regular habits of life, and the avoidance of cancer subjects by those predisposed.

